# The impact of shared knowledge on speakers’ prosody

**DOI:** 10.1371/journal.pone.0223640

**Published:** 2019-10-14

**Authors:** Amandine Michelas, Cécile Cau, Maud Champagne-Lavau

**Affiliations:** Aix Marseille Univ, CNRS, LPL, Aix-en-Provence, France; Abertay University, UNITED KINGDOM

## Abstract

How does the knowledge shared by interlocutors during interaction modify the way speakers speak? Specifically, how does prosody change when speakers know that their addressees do not share the same knowledge as them? We studied these effects in an interactive paradigm in which French speakers gave instructions to addressees about where to place a cross between different objects (e.g., You put the cross between the red mouse and the red house). We manipulated (i) whether the two interlocutors shared or did not necessarily share the same objects and (ii) the informational status of referents. We were interested in two types of prosodic variations: global prosodic variations that affect entire utterances (i.e., pitch range and speech rate variations) and more local prosodic variations that encode informational status of referents (i.e., prosodic phrasing for French). We found that participants spoke more slowly and with larger pitch excursions in the not-shared knowledge condition than in the shared knowledge condition while they did not prosodically encode the informational status of referents regardless of the knowledge condition. Results demonstrated that speakers kept track of what the addressee knew, and that they adapted their global prosody to their interlocutors. This made the task too cognitively demanding to allow the prosodic encoding of the informational status of referents. Our findings are in line with the idea that complex reasoning usually implicated in constructing a model of the addressee co-exists with speaker-internal constraints such as cognitive load to affect speaker’s prosody during interaction.

## Introduction

The way speakers share or do not share knowledge with their interlocutors during interaction has consequences for words they produce but also for the prosody associated with the words. The term *prosody* refers to variations of suprasegmental features of speech including pitch, duration and intensity. When these three acoustic parameters vary, it induces different kinds of prosodic variations. First, it creates local prosodic variations that affect words or small groups of words through prosodic prominence attribution (i.e., through the degree to which these words perceptually stand out from surrounding words). Second, it provides more global prosodic variation that affects entire utterances such as speech rate and pitch range variations. The present article deals with the possible impact of shared knowledge manipulation between interlocutors on one or both of these two kinds of prosodic variations in French.

The first evidence of the impact of shared knowledge on speakers’ prosody comes from the fact that speakers encode the already-mentioned vs. contrastive status of words through prosodic prominence by consulting information that is mutually shared with their interlocutors ([1]). For instance, in most Germanic languages such as English or Dutch, speakers select deaccenting forms for already-mentioned words and prosodic prominent forms for contrastive words ([1–5]). In contrast to Germanic languages, it appears that the correspondence between deaccenting vs. prosodic prominence and information status is less systematic in Romance languages. For instance, it appears that Catalan and Spanish make less use of prosodic prominence to mark information status but also rely on syntactic strategies affecting word order (see [6], for a discussion of the distinction between the prosodic marking of information status in Germanic and in Romance languages). Similar to what happens in Catalan and Spanish, at least two strategies are allowed in French to encode the new vs. given status of words: syntactic strategies such as dislocations (see [7]) or just prosodic marking. In French, due to the absence of lexical stress, the prosodic encoding of focus is different from that one of many other Romance languages, and mainly relies on prosodic phrasing (i.e., the grouping of words into prosodic units of different sizes). Specifically, a number of studies have reported a tendency for contrastive words to be parsed in a separate prosodic unit from the rest of the utterance ([7–11]). For instance, using a paradigm in which participants had to correct sentences produced by a fictitious interlocutor (a prompt), Dohen & Loevenbruck [8] showed that participants produced utterances with the verb phrase and the object noun phrase grouped together in the same prosodic constituent (the Accentual Phrase or AP) in contexts in which no element of the utterance contrasted with a previously-mentioned discourse element (e.g., *[Les loups]*_*AP*_ *[suivaient Marie-Lou]*_AP_ ‘The wolves were following Marie-Lou’). However, they produced the element that contrasted with a previously mispronounced element in a separate AP when they knew that the prompt conveyed false-belief about this element (e.g., [Les loups]_AP_ [suivaient]_AP_ [Marie-Lou]_AP_ when ‘Marie-Lou’ was mispronounced). More recently, in an interactive paradigm in which a director had to indicate noun-adjective pairs of items to an addressee, Michelas, Faget, Portes, Lienhart, Boyer, Lançon, et al. ([11]), showed that French participants parsed the target noun in the same AP as the following adjective when it was identical to the noun in the preceding fragment (e.g., *bonbons marron* ‘brown candies’ followed by [*bonbons violets*]_AP_ ‘purple candies’) while they parsed it in a separate AP from the following adjective when it contrasted with it to warn their interlocutor that this noun constituted a contrastive entity (e.g., *bougies violettes* ‘purple candles’ followed by [*BONBONS*]_AP_[*violets*]_AP_ ‘purple candies’). It is not clear whether this phenomenon resulted from a speakers’ willingness to help their addressee (as predicted by ‘the audience design hypothesis’; see [12]) or whether it is mediated by production-internal constraints on the speakers’ part that favor speech that is easy to produce ([13–17]). Nevertheless, it appears that when speakers share a set of alternatives with their partner, they prosodically encode the new vs. given status of word.

Moreover, it is possible that the way in which interlocutors shared knowledge during the interaction affects not only the prominent words but also all other regions of speakers’ utterance. This idea is related to the fact that when speakers are aware of a speech perception difficulty on the part of addressees, they modify their global prosody (see [12] for a review). For instance, a lot of studies focusing on ‘clear speech’ (a distinct listener-oriented speaking style that is more intelligible than conversational speech) converge on the idea that speakers decrease their speech rate and increase their wider dynamic pitch range in order to accommodate addressees in contexts in which their listener experience background noise, hearing impairments or a different native language, ([18–26]). Following this idea, it is reasonable to hypothesize that when speakers are aware they do not share the same knowledge as their interlocutors, they produce clear speech thus modifying their global prosody to prevent their listeners from comprehension difficulties.

In the present study, we collected speech samples in a controlled but interactive paradigm that enabled us to measure prosodic variations on speech fragments that refer either to objects shared by the two interlocutors (***shared knowledge condition)*** or that refer to objects that might differ between the two interlocutors ***(not-shared knowledge condition***). In the paradigm we built, participants played an interactive game. They were seated in a quiet room with a confederate, facing each other, both with a computer screen. The participants had to give instructions to the confederate about where to place a cross between the different objects displayed on the screen (e.g., *Tu mets la croix entre la souris bordeau et la maison bordeau*; ‘You put the cross between the red mouse and the red house’). The participants knew that when objects appeared in white boxes, the confederate shared exactly the same objects as them on her screen while this was not necessarily the case when objects appeared in black boxes. In this case, participants knew that objects displayed on black boxes could differ either in their type (e.g., mouse vs. car) or in their color (e.g., red vs. purple) on the confederate’s screen. We had two goals. Our first goal was to test whether the fact that the speakers knew their addressee shared or did not necessarily share the same set of objects/colors as them, affected the global prosodic variations they produce. Following the idea that speakers adapt their global prosody to the difficulties encountered by their addressee during spoken interaction, we hypothesized that, in the absence of shared knowledge between participants, speakers would modify their global prosodic features such as speech rate and pitch range to facilitate addressees’ comprehension as it is the case in clear speech. Specifically, we predicted that speakers would speak more slowly and with larger pitch incursions in a condition in which they knew their interlocutor did not necessarily share the same knowledge (***not-shared knowledge condition***) compared to a condition in which they knew s/he shared it (***shared-knowledge condition***). Our second goal was to test whether shared knowledge between interlocutors affected prosodic variations that encoded informational status of referents. To do so, we manipulated the contrastive status of target fragments that always appeared in second position within pairs produced by the speaker (e.g., *maison bordeau* ‘red house’ in *Tu mets la croix entre la souris bordeau et la maison bordeau*; ‘You put the cross between the red mouse and the red house’). Within these target fragments, (1) the target noun was identical to the noun of the 1^st^ fragment while the target adjective contrasted to the one in the 1^st^ fragment (e.g., *maison violette* vs. *maison BORDEAU)* ***contrastive adjective***, (2) the target noun in the 2^nd^ fragment contrasted to the noun in the 1^st^ fragment while the adjective was identical (e.g., *souris bordeau* vs. *MAISON bordeau*; ***contrastive noun***) (3) both the target noun and the target adjective contrasted to their counterparts in the 1^st^ fragment (e.g., *souris violette* vs. *MAISON BORDEAU*; ***contrastive fragment***). Following Michelas, Faget, Portes, Lienhart, Boyer, Lançon, et al. ([11]) and others, we hypothesized that the presence of shared knowledge between participants would affect the prosodic encoding of informational status of referents and thus the prosodic phrasing produced by French participants. Specifically, we predicted that speakers would isolate contrastive nouns in separate APs when they knew their interlocutor shared exactly the same set of alternatives/objects as them (***shared knowledge condition***) but not when they knew their interlocutor did not necessarily share it (***not-shared knowledge condition***). Thus, we expected participants to produce more 2 AP phrasing in the contrastive noun condition (*souris bordeau vs*. *MAISON bordeau*, ‘red mouse vs. red house’) compared to the contrastive adjective condition (maison violette vs. maison BORDEAU ‘purple house vs. red house’) in shared knowledge circumstances but not in not-shared knowledge circumstances. In this last case, it would not be relevant for speakers to highlight one particular alternative to their addressee since they knew their interlocutor did not necessarily share the same set of alternatives as them. Finally, we tested an additional condition in which both the target noun and the following adjective were contrastive (*souris violette vs*. *MAISON BORDEAU* ‘purple mouse vs. red house’) to see whether our chance to obtain a 2 AP phrasing is increased when the entire fragment (and not only the noun) is contrastive. This hypothesis is based on Michelas, Faget, Portes, Lienhart, Boyer, Lançon, et al. ([11])’s study who obtained more 2 APs phrasing (72% of cases) than 1 AP phrasing (28% of cases) on filler items showing this kind of “double contrast”. If this double contrast actually favors the parsing of the noun in a separate AP from the following adjective as it was the case in Michelas, Faget, Portes, Lienhart, Boyer, Lançon, et al. ([11])’s study, speakers would produce more 2 APs phrasing in our contrastive fragment condition compared to our contrastive noun condition in shared knowledge circumstances.

## Method

### Participants

Twenty-two participants (16 women) between 18 and 47 years old (mean age = 21.4, SD = 5.9; mean education: 13.8 years, SD = 0.8) participated in the experiment. All participants gave written informed consent. All reported having no neurological, psychiatric, visual or hearing impairment. They were all native speakers of French, the language of the experiment. The study was approved by the university’s ethics review board (*Comité d’évaluation éthique d’Aix-Marseille université*) and conducted according to the principles expressed in the Declaration of Helsinki. The experiment was classified as purely behavioral, and the testing involved no discomfort or distress to the participants.

### Materials

**Fig 1:**
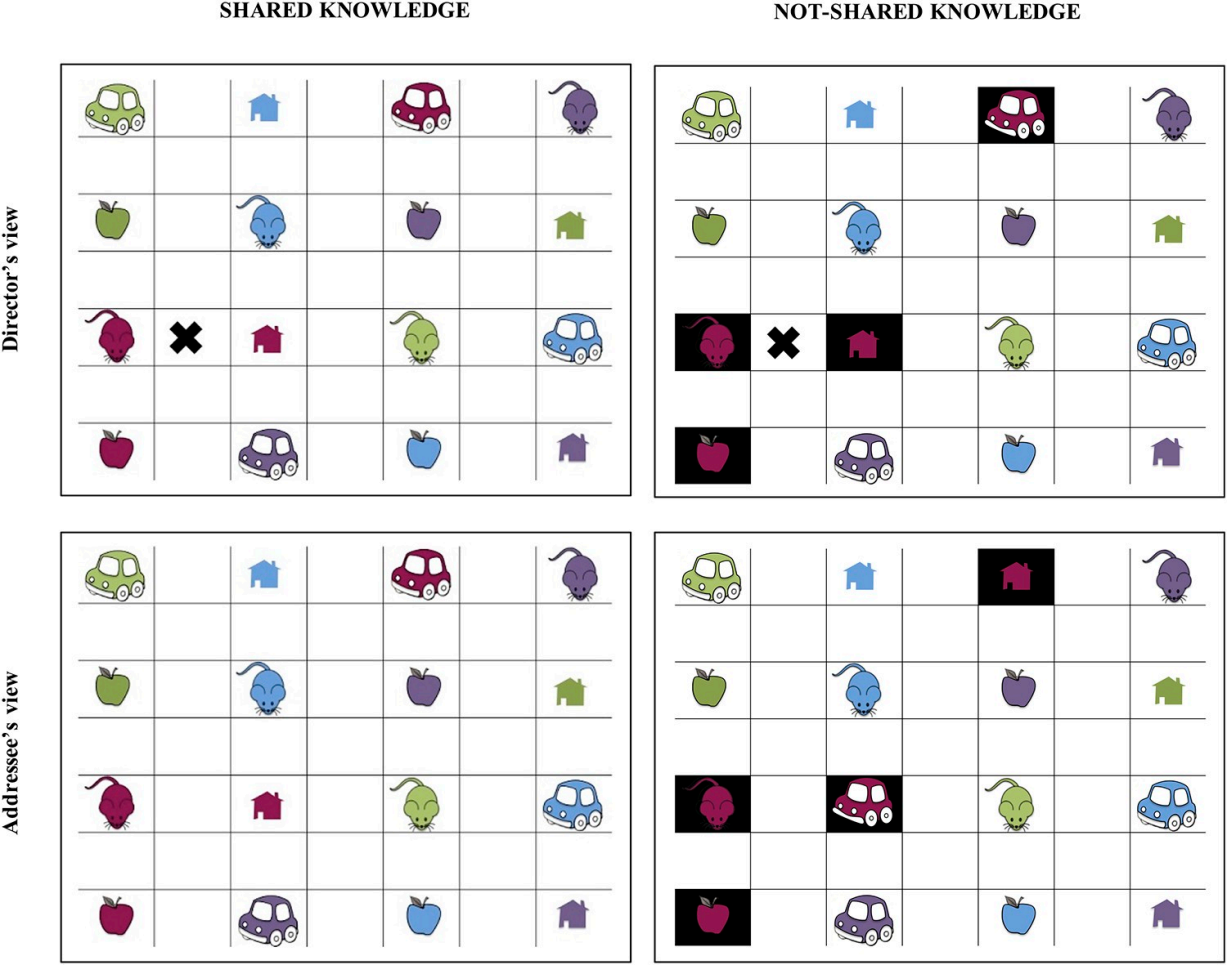
Director and addressee’s views for the *maison bordeau* ‘red house’ fragment in the contrastive noun shared knowledge condition (left panel) and not-shared knowledge condition (right panel). The director’s view includes the cross (top panel) while the addressee’s view does not (bottom panel). This figure is similar but not identical to the original image published in Michelas, Cau & Champagne-Lavau [27]) and is therefore for illustrative purposes only.

An interactive task involving cooperation between a director (the participant) and an addressee (a confederate) was developed for the present study. During this task, the director had to give instructions to the addressee about where to place a cross between different objects on a grid by naming the type and the color of two critical objects (e.g., *Tu mets la croix entre la souris bordeau et la maison bordeau*; ‘You put the cross between the red mouse and the red house’). Each grid was composed of 16 objects corresponding to the combination of 4 types of objects and 4 colors. For instance Fig 1 illustrates the director’s and addressee’s grid built around the 4 following colors: *bordeau* ‘red’ (critical color), *violet* ‘purple’ (critical color), *vert* ‘green’ (filler color), *bleu* ‘blue’ (filler color) and the 4 types of object: *souris* ‘mouse’ (critical object), *maison* ‘house’ (critical object), *pomme* ‘apple’ (filler object), voiture *‘car’* (filler object). A total of 56 different types of object were used to build the 84 grids of the experiment. These grids were created from 14 target fragments (2 knowledge conditions x 3 informational status conditions x 14 target noun-adjective fragments). The objects and the colors of the critical pair were chosen so that we controlled for the words produced by the participants (e.g., the number of syllables, the syllable structure and the phonemic characteristics of words).

In half of the grids, the cross has to be placed horizontally (as it is the case in Fig 1) whereas in the other half, it has to be placed vertically. The location of the cross was stable for the same target fragment (e.g. maison bordeau ‘red house’) that appeared six times in the six different conditions (see details of the 6 experimental conditions below) but was never the same across the 14 target fragments so that participants cannot anticipate the location of the cross. In the immediate proximity of the two target items (i.e., below the two target items when the cross has to be placed horizontally and on the left of the two target items when the cross has to be placed vertically), a competitor object and a filler object were always visible. The competitor object consisted of a different object of the same color for the ***contrastive noun condition*** (see the list of the experimental conditions below and in Table 1), the same object but in a different color for the ***contrastive adjective condition*** and a different object with a different color for the ***contrastive fragment condition***. The filler object was always a different object than the objects used for the target pair and the competitor and had always a different color. The other objects on the grid were randomly arranged. Each pair of target items gave rise to six different grids (one for each experimental conditions). The arrangement of objects was the same for each sextuplet of grids except for the white/black boxes manipulations as explained in the following paragraph.

**Table 1:** Noun-adjective pairs, corresponding to the *maison bordeau* ‘red house’ target fragment, elicited by the task and thus produced by directors for each of the 6 experimental conditions.

	Shared knowledge	Not-shared knowledge
	*Directors knew addressees shared the same object/color*	*Directors knew* addressees *did not necessarily share the same object/color*
**Contrastive adjective**	maison violette/maison bordeau	maison violette/maison bordeau
	'purple house/red house'	'purple house/red house'
**Contrastive noun**	souris bordeau/maison bordeau	souris bordeau/maison bordeau
	'red mouse/red house'	'red mouse/red house'
**Contrastive fragment**	souris violette/maison bordeau	souris violette/maison bordeau
	'purple mouse/red house'	'purple mouse/red house'

We developed two experimental manipulations. First, to control for the presence vs. absence of shared knowledge between participants, we manipulated the speaker’s knowledge about whether his/her addressee shared or did not necessarily share the same objects as him/her. To do so, in the ***not-shared knowledge condition***, target items appeared in black boxes and participants knew that they could have different objects or different colors for objects in those boxes (see Fig 1 bottom panel) while in the ***shared knowledge condition***, items appeared in white boxes meaning for the participants that their interlocutor shared exactly the same objects as him/her (see Fig 1 top panel). The black boxes were always four in number and their distribution was conditioned by the type of contrast for the current pair of objects. For instance, for the ‘red mouse vs. red house’ pair (contrastive noun), the four black boxes affected the four red objects. In the same manner, for the ‘purple house vs. red house’ pair (contrastive adjective), the four black boxes affected the four houses. For the contrastive fragment pair, the four black boxes affected the four objects of the same color as the target item for half of the target pairs (e.g., the four red objects for the ‘purple mouse vs. red house’ pair) or the four objects of the same type as the target item for the other half of the target pairs (e.g., the four mills for the ‘orange sun vs. brown mill’ pair). To obtain the addressee’s grid in the not-shared knowledge condition, the second object of pairs of objects was always replaced by one of the two other possible alternatives on the grid. For instance, in Fig 1, the red house in the director’s grid was replaced by the red car in the addressee’s grid. This manipulation allowed us to create a situation in which participants always shared the same set of objects for a given grid but knew that, sometimes, critical objects or colors might differ at a given location on the addressee’s screen and sometimes they might actually be the same even though they appeared in black boxes. The location of black boxes was stable for the same target fragment that appeared six times in the six different conditions but was different across the 14 target fragments so that participants cannot anticipate their location. The complete instructions given to participants are shown in S1 Appendix.

Second, in order to control for the informational status of referents, we manipulated whether the noun or the adjective in the 2^nd^ fragment was the same or contrasted with the noun or the adjective in the 1^st^ fragment. We obtained three types of informational status: (1) the noun in the 2^nd^ fragment was kept constant while the adjective in the 2^nd^ fragment contrasted to the adjective in the 1^st^ fragment (e.g., *maison violette* vs. *maison BORDEAU)* ***contrastive adjective*** (2) the noun in the 2^nd^ fragment contrasted to the noun in the 1^st^ fragment while the adjective was kept constant (e.g., *souris bordeau* vs. *MAISON bordeau*; ***contrastive noun***) (3) both the noun in the 2^nd^ fragment contrasted to the noun in the 1^st^ fragment and the adjective in the 2^nd^ fragment contrasted to the adjective in the 1^st^ fragment (e.g., *souris violette* vs. *MAISON BORDEAU*; ***contrastive fragment***).

The combination of the two experimental factors (type of knowledge and informational status of target fragment) led to 6 experimental conditions. The 6 noun-adjective pairs corresponding to the 6 experimental conditions for the *maison bordeau* target fragment were given as example in Table 1. The target fragment was always the second fragment of the pair (e.g., red house in the ‘red mouse vs. red house’ pair). Each participant saw the 84 screens (2 knowledge conditions x 3 informational status conditions x 14 target noun-adjective fragments) that appeared simultaneously on his/her computer’s screen and on the computer’s screen of his/her addressee. The order of presentation of screen view was fixed across participants but was semi-randomized within participants so that the same target item did not appear more than twice in succession and the same experimental condition was not repeated more than twice in a row.

### Procedure

The director and the addressee were seated, facing each other, both with a computer screen. They could not see each other’s computer screen. The director was asked to give instructions to the addressee about where to place the cross by indicating to the addressee the type and the color of objects (e.g., *Tu mets la croix entre la souris bordeau et la maison bordeau*; ‘You put the cross between the red mouse and the red house’). The addressee was always the same confederate (i.e., a native French female speaker). She was introduced to the participant as the experimenter but was naïve about the scientific aims of the experiment. However, since the addressee performed the task 22 times in the exact same order, she received instructions about the feedbacks she was allowed to produce. These instructions were intended to homogenize the feedbacks she produced from one participant to another. When she interacted with the participant, she was explicitly asked to give him/her continual feedback of two types. First, in cases in which she identified immediately and without effort where to put the cross, she was asked to signal understanding with feedbacks such as *d’accord* ‘okay’, *ok* ‘okay’, *c’est bon* ‘*I’m good’*. Second, in cases in which she had difficulties to identify where to place the cross (typically in case of black boxes), she was instructed to tell the participant why she is in difficulties (e.g., *Alors je n’ai pas de souris bordeau et de maison bordeau à côté* ‘Well, I don’t have any red house next to a red mouse’) and to ask them for clarifications leading to several exchanges between the two interactional partners. Clarification questions could be of three types: (i) vague clarification question (e.g., *Je n’ai pas bien compris* ‘I don’t understand very well’, *Tu peux répéter*? ‘Can you repeat?’, *Je suis un perdue*, *est-ce que tu peux me répéter ce que tu vois* ? ‘I’m lost, can you tell me what you see ? ’), (ii) precise clarification question (e.g., *Où est-ce que je dois mettre la croix par rapport à la souris bordeau* ? ‘Where should I put the cross in relation to the red mouse?’, *Dans quelle colonne se situe la croix*? ‘In which column is the cross located ? ’) (iii) inference clarification question (e.g., *Est-ce que je dois mettre la croix en dessous du bureau bordeau*? ‘Do I have to put the cross below the red desk ? *Est-ce que la croix est à gauche du crayon marron* ? ‘Is the cross to the left of the brown pencil ?’, *Est-ce que la croix est sur la même ligne que le crayon violet* ? ‘Is the cross on the same line as the purple pencil?’). After identifying the location of the cross, the confederate had to show the participant her understanding using feedback such as ‘okay’. We recorded both the director’s and addressee’s productions, but we only analyzed the director’s productions. The types of feedback did not give rise to any coding. Moreover, regardless of the type of exchange that took place between the director and the addressee, the segment of speech we analyzed was always extracted from the initial production of the speaker. This segment was elicited before any confederate’s reaction. For instance, we did not analyze cases in which the speaker repeated target fragments after a clarification question asked by the addressee. An extract from the dialogue obtained between one participant and the confederate is given as example in S2 Appendix. This extract corresponds to the screen views shown in Fig 1.

Each screen view appeared simultaneously on the director and the addressee computer’s screen. Once the addressee has moved the cross with the computer mouse on his/her screen, both participants were asked to simultaneously click on the space bar to make the next screen view appeared. The director and the addressee were recorded in a quiet room using a Zoom H4N Handy Recorder and a Headset Cardioid Condenser Microphone (AKG C520). We analyzed directors’ speech only.

The experimental phase began with 6 practice screen views followed by 84 target screen views. The 6 practice screen views were built from 8 new objects and 8 new colors that did not appear in the 84 target screen views.

To ensure that directors could identify and use a consistent label for each target object and color, before the experimental phase, participants performed a familiarization phase during which they had to report aloud what they saw in 64 pictures. These 64 pictures were composed of all the possible objects participants met in the experimental phase (56 different objects) in one of the 4 possible colors used for the experimental phase plus the 8 objects of the 6 practice items also randomly assigned to one of the possible colors. When participants mispronounced or use a different labels to name the target objects and/or the target colors, they were corrected by the experimenter, they were asked to repeat the correct labels and to remember these labels, as they would see the same object/color in the next phase of the game.

### Measures and annotations

One item that gave rise to object label errors in more than 75% was removed from the analyses. Analyses were performed on pairs containing neither disfluencies/hesitations nor object label errors for a total of 1461 pairs, thus removing 255 pairs (14.9%) out of the 1716 pairs. In these pairs, we analyzed both global prosodic variations and prosodic phrasing as a marker of informational status of referents.

**Fig 2:**
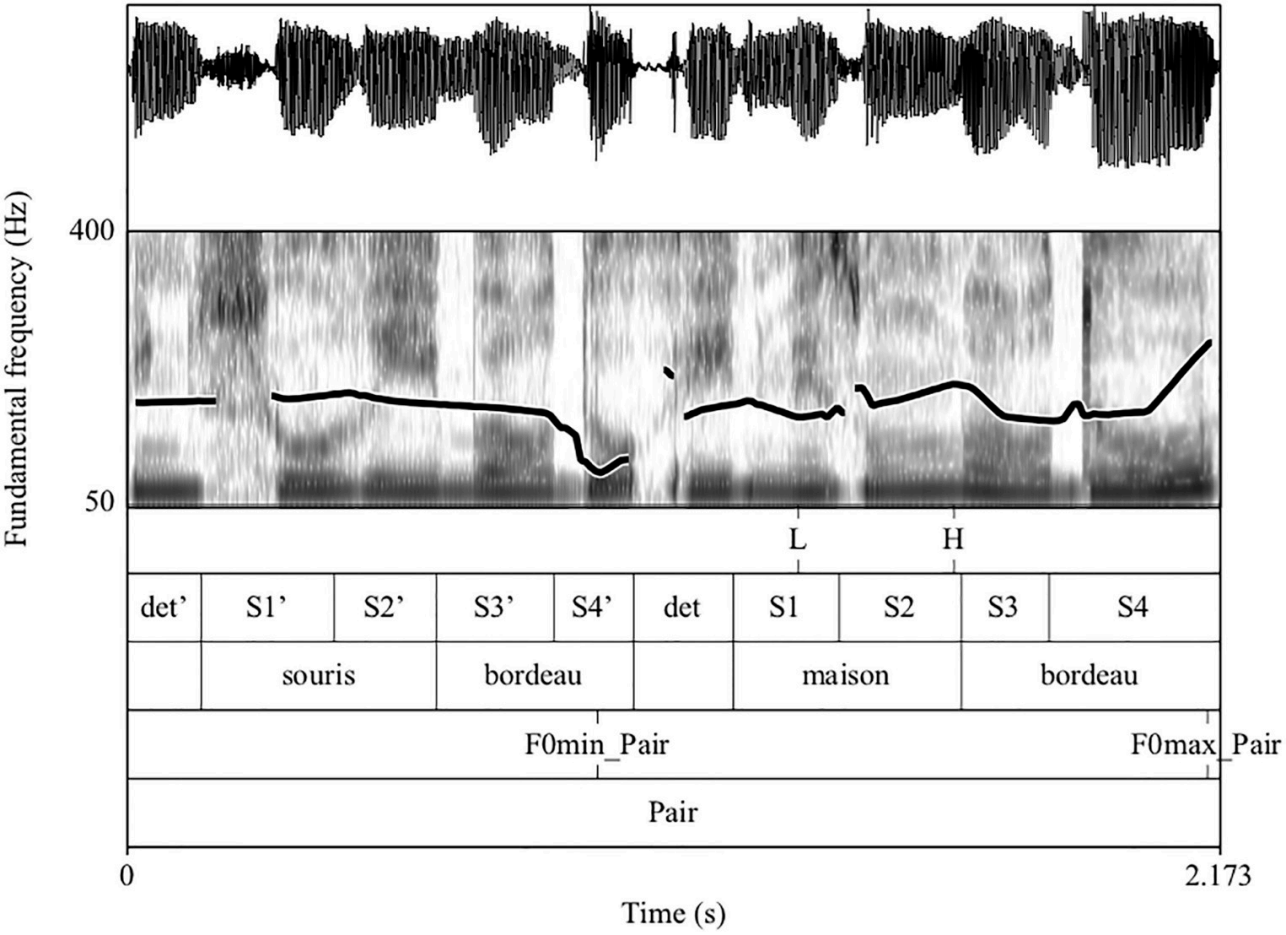
Schema annotation for the pair *une souris bordeau et une maison bordeau* ‘the red house and the red house’.

We first analyzed whether speakers modulate their global prosody depending on whether they shared knowledge or not with their interlocutor. To do so, we measured speech rate and pitch range. These are the two main global prosodic acoustic-phonetic features that characterize clear speech production (see [12]). We calculated average speech rate in syllables/second for the entire pairs of noun-adjective fragments (e.g., ‘the red mouse and the red house’). We first calculated the total duration of the entire pair of noun-adjective fragments (see ‘Pair’ in Fig 2) according to the following procedure. Each entire pair of noun-adjective fragments (e.g., *la souris bordeau et la maison bordeau* ‘the red mouse and the red house)’ had a base syllable count of 11 syllables. The first author listened to each utterance and adjusted the base count for the very rare cases in which the speaker did not produce the schwa at the end of the article *le* ‘the’ (e.g. [lə. mu. tɔ̃. ma. ʁɔ̃. e. **lbe**. ʀɛ. ma. ʁɔ̃] instead of [lə. mu. tɔ̃. ma. ʁɔ̃. e. **l**ə**. be**. ʀɛ. ma. ʁɔ̃] *le mouton marron et le beret marron* ‘brown sheep and brown beret’). This syllable count was divided by the utterance duration to obtain a rate measurement for each entire pairs of noun-adjective fragments. We then measured pitch range variations. Two main types of pitch range variations have been reported in the literature: the first is called ‘pitch level’ ([28]) or ‘register’ ([5]) and corresponds to the raising or lowering of the melodic movements in the fundamental frequency (F0) space. For instance, when we speak with higher pitch level than usual to imitate the voice of a child, both F0 minima and maxima are produced higher so that the entire F0 contour is produced higher in the F0 space. The other way in which pitch range is varied concerns the ‘pitch span’ ([28]) also referred to as the ‘excursion size’ ([29]). This measure refers to the distance between the highest and the lowest pitches point in the contour. When the pitch span is increased, F0 maxima are produced higher than usual while F0 minima stay the same. Since clear speech research has reported ‘a wider dynamic pitch range’ (e.g., a larger pitch span) when speakers are aware of perception difficulties from their listener, we measured pitch range variations as pitch span variations in our study. We defined pitch span as the difference between maximum and minimum F0 values for the entire pair of noun-adjective fragments. Maximum and minimum F0 values were automatically extracted using Praat scripts ([30]).

Second, in order to analyze whether the presence of shared knowledge between participants affected the prosodic encoding of informational status of referents, we examined whether speakers produced the noun in the 2^nd^ fragment in the same AP as the following adjective (e.g., [*maison bordeau*]_AP_ ‘red house’) or in a separate AP (e.g., [*maison*]_AP_ [*bordeau*]_AP_). Pre-boundary lengthening and the presence of a typical F0 rise aligned with the last syllable of the AP are well known as the two main correlates of AP right boundaries in French ([31–33]). Specifically, the last full syllable of the AP has been shown to exhibit longer duration than unaccented syllables within an AP. In addition, APs in non-final position within the utterance are typically characterized by a rising F0 contour aligned with the last full syllable of the AP. For these reasons, we measured duration of the first and second syllables of target nouns (respectively S1 and S2 in Fig 2). We also extracted F0 values corresponding to the F0 minimum hertz value of the 1^st^ syllable of target nouns and to the F0 maximum hertz value of the last syllable of target nouns. Based on these tagged features, we used two specific criteria to define the presence of an AP right boundary (see Michelas, Faget, Portes, Lienhart, Boyer, Lançon, et al. ([11) for the same procedure). First, following the pitch accent detection criterion used by [34] on French data, we considered that an AP right boundary was actually produced by the speaker after the target noun if the F0 maximum hertz value of the last syllable of the target noun was at least 10% higher than that of the F0 minimum hertz value of the 1^st^ syllable of target nouns. Second, we also verified that the syllable was lengthened. To do so, we checked whether the duration of the last syllable of the target noun was at least 10% longer than that of the preceding syllable. This is in line with Michelas, Faget, Portes, Lienhart, Boyer, Lançon, et al. ([11) who found that healthy control speakers parsed contrastive referents in a separate AP using two specific criteria to define the presence of an AP boundary (a 10% threshold for pitch accent detection and a 10% threshold for syllable lengthening). [33] reports results from a corpus study that compared vowel duration in different prosodic positions (within words, in AP-final position, in ip-final position and in IP-final position) at different speech rates (i.e., normal and fast speech rates) and for different speakers (i.e., males and females). This study also demonstrated that AP-final vowels are more or less 10% longer than a vowel contained within an AP and is thus in accordance with the criterion used by [33].

All the performed annotations are summed up below and illustrated in Fig 2:

**Duration measures:**
**Pair**: entire noun-adjective pair
**det’**: determinant of the 1^st^ fragment
**S1’**: 1^st^ syllable of the 1^st^ fragment
**S2’**: 2^nd^ syllable of the 1^st^ fragment
**S3’**: 3^rd^ syllable of the 1^st^ fragment
**S4**’: 4^th^ syllable of the 1^st^ fragment
**det**: determinant of the 2^nd^ fragment
**S1**: 1^st^ syllable of the 2^nd^ fragment
**S2**: 2^nd^ syllable of the 2^nd^ fragment
**S3**: 3^rd^ syllable of the 2^nd^ fragment
**S4**: 4^th^ syllable of the 2^nd^ fragment

**F0 measures:**
**F0min_Pair:** F0 minima for the entire pair of fragments
**F0max_Pair:** F0 maxima for the entire pair of fragments
**L:** low inflection point in the F0 curve near the beginning of the first syllable of the noun in the 2nd fragment
**H:** F0 maximum hertz value of the last syllable of the same target noun

All the values corresponding to these labels were extracted thanks to Praat ([30]) scripts written by our own.

## Results

### Global prosodic variations

**Fig 3:**
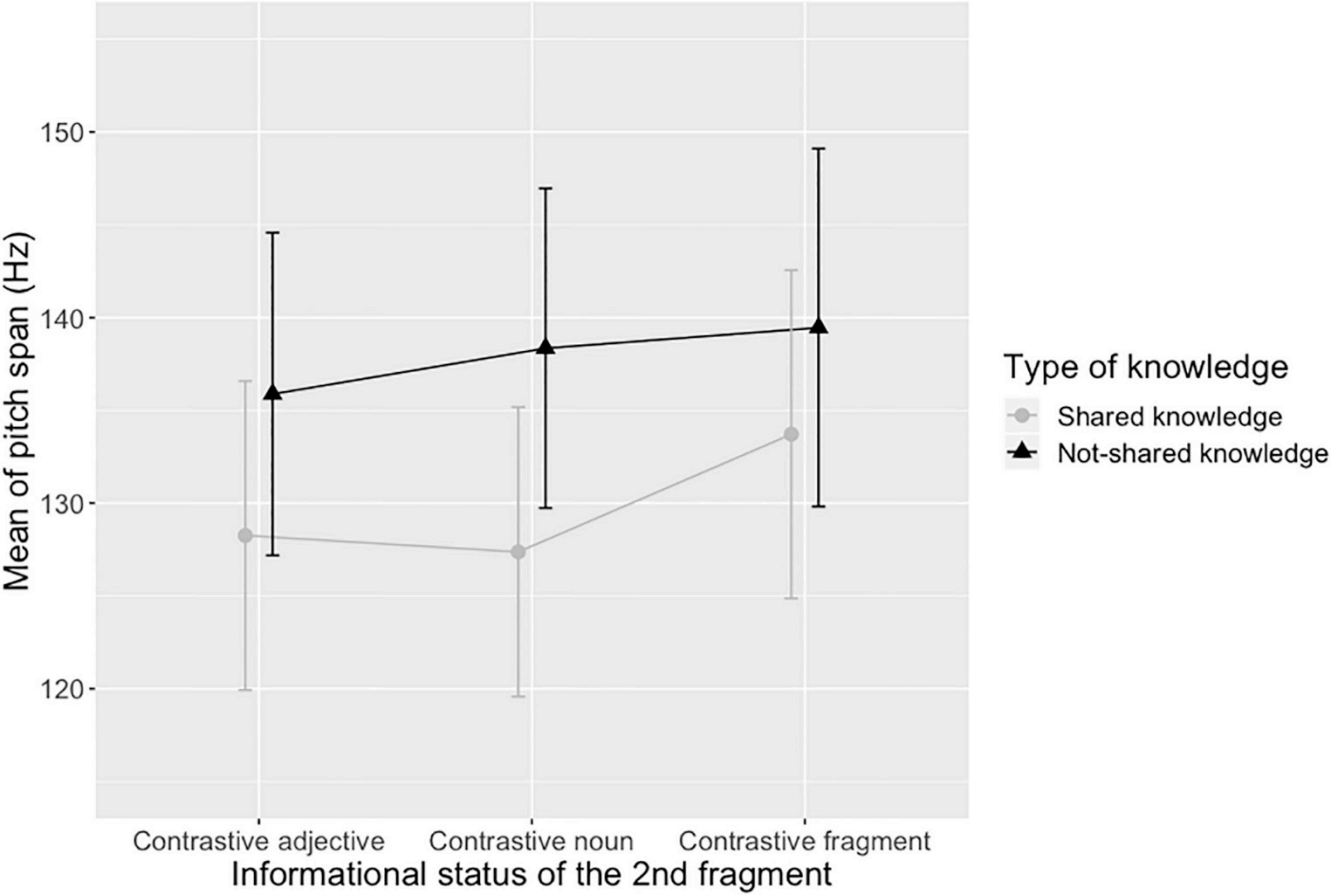
Mean of pitch span depending on the type of knowledge and the informational status of the target fragment. Error bars show 95% confidence intervals.

**Fig 4:**
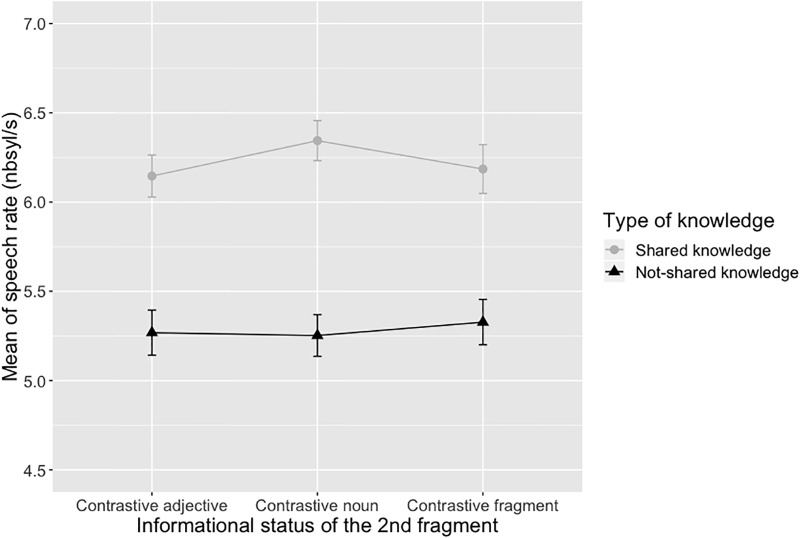
Mean of speech rate depending on the type of knowledge and the informational status of the target fragment. Error bars show a 95% confidence interval.

Mean of pitch span (in Hz) and mean of speech rate (in syl/s) are shown in Figs 3 and 4 respectively.

Two linear mixed-effects regression models were used on the log-transformed pitch span and speech rate values. Taking the log transformations ensures a normal distribution of the residuals ([35]). Both models included the informational status, the type of knowledge and the interaction between the two factors as fixed effects. Following [36], we used the maximal random effects structure that allowed the model to converge. Participants and items were thus included as random intercepts (random slopes by participant for the effect of informational status and type of knowledge were included only for the speech rate model since they did not allow the pitch span model to converge). Global effects were obtained using the afex::mixed function.

The pitch span model revealed a significant main effect of type of knowledge (*X*^2^ = 8.65 p < .01) due to a larger pitch span in the not-shared knowledge condition compared the shared-knowledge condition. The effect of informational status (*X*^2^ = 3.07, p >.20) and the informational status x type of knowledge interaction (*X*^2^ = 3.90, p = .14) were not significant. Since referents were repeated 6 times across our 6 experimental conditions and since repetition is a well-established contributor to acoustic attenuation, we reran the same model with repetition as a fixed factor. Repetition was coded R1-R6 and was not centered because it was not a numerical variable. This additional model showed that there was no significant effect of the repetition factor (*X*^2^ = 2.69, p >.20).

The speech rate model revealed a significant main effect of type of knowledge (*X*^2^ = 37.37, p < .0001) due to faster speech rate in the shared knowledge condition compared to the not-shared knowledge condition. The effect of informational status was not significant (*X*^2^ = 4.25, p = .12). The informational status x type of knowledge interaction was also significant (*X*^2^ = 7.60, p < .05). Multiple comparisons (Tukey test, p <. 05) obtained with the glht function from the multcomp package ([37]) with p-values adjusted by the single step method revealed that this significant interaction was due to faster speech rate in the contrastive noun condition compared to the contrastive fragment condition (z = 3.00, p < .05) and a tendency for faster speech rate in the contrastive noun condition compared to the contrastive adjective condition (z = 2.81, p = 0.056) in the shared knowledge condition. As for the pitch span model, we reran our statistical model with repetition as a fixed factor. In a similar way to what happened for the pitch span model, there was no significant effect of repetition on the log-transformed speech rate values (*X*^2^ = 4.66, p >.20).

### Prosodic encoding of informational status

Proportion of items in the coded sample that included 2 APs depending on the type knowledge shared by participants and the informational status of target fragments shared is shown in Table 2.

**Table 2:** Proportion of items in the coded sample that included 2 APs phrasing (in %).

	Type of knowledge	
	Shared knowledge	Not-shared knowledge
Contrastive adjective	20	25
Contrastive noun	24	21
Contrastive fragment	21	20

As shown in Table 2, there was a large bias in favor of the 1 AP phrasing compared to the 2 AP phrasing regardless of the type of knowledge and of the informational status of target fragments. We used a mixed-effects regression model (lme4 package in R-studio statistics Version 0.99.903) with a logistic linking function to confirm the statistical relevance of this bias. The model included the AP phrasing as dependent variable (1 = 2 APs, 0 = 1AP), the informational status (contrastive adjective, contrastive noun, contrastive fragment), the type of knowledge (shared-knowledge, not-shared knowledge) and the interaction between the two factors as fixed effects. Following [36], we used the maximal random effects structure that allowed the model to converge. Participants and items were thus included as random intercepts only (random slopes by participants and by items were not included because they did not allow the model to converge). Neither the effect of the two factors (effect of informational status: *X*^2^ = 0.24, p > .20; effect of type of knowledge: *X*^2^ = 0.0004, p > .20), nor the informational status x type of knowledge interaction was significant (*X*^2^ = 2.00, p > .20). As with the global prosodic variations, we reran our statistical model with repetition as fixed factor. The additional model showed that there was no significant effect of repetition on the AP-phrasing produced by participant (*X*^2^ = 3.19, p >.20).

**Fig 5:**
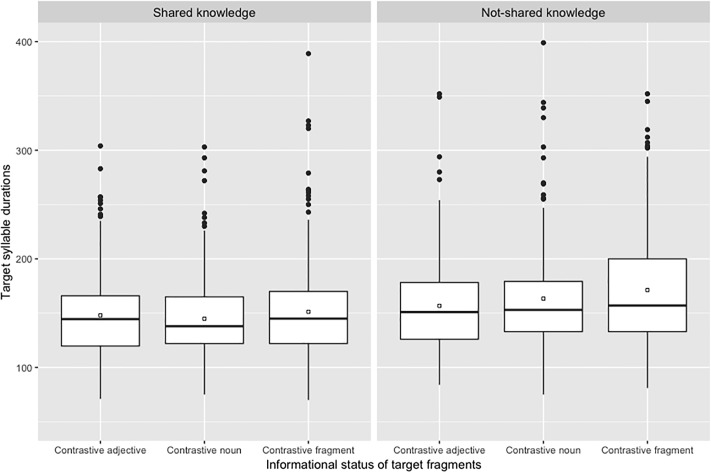
Boxplots of target syllable duration (in ms) depending on the informational status of target fragments and knowledge shared by participants.

**Fig 6:**
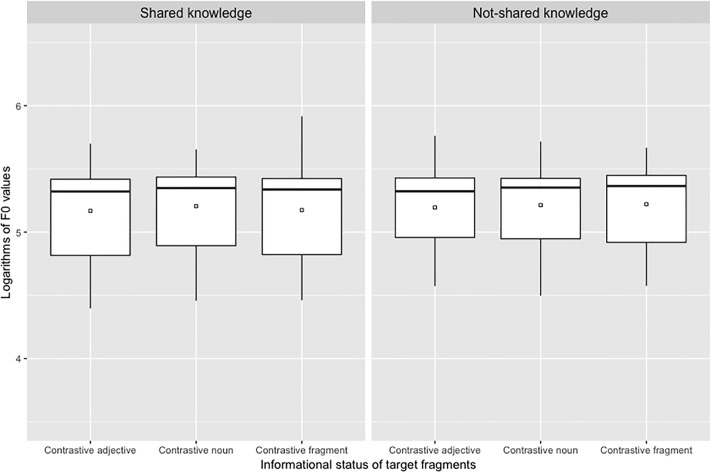
Boxplots of logarithms of maximum F0 values associated with target syllables depending on the informational status of target fragments and knowledge shared by participants.

We also fitted two linear mixed effects models on the acoustic parameters we used (duration and F0 values associated with target syllables) to define the presence or the absence of an AP at the right edge of target noun. Durations of target syllables in milliseconds (S2’ in Fig 2 above) are illustrated in Fig 5 while logarithms of F0 maxima associated with target syllables (H in Fig 2 above) are illustrated in Fig 6.

The two models included log-transformed values as dependent variables to ensure a normal distribution of the residuals ([35]). The informational status of referents (contrastive adjective, contrastive noun, contrastive fragment), the type of knowledge (shared-knowledge, not-shared knowledge) and the interaction between the two factors were included as fixed effects. Following [36], we used the maximal random effects structure that allowed the model to converge. The duration values model also included random intercepts by participant and by item and random slopes by participant for the two fixed effects. For the F0 values model, participants and items were included as random intercepts only (random slopes by participants and by items were not included because they did not allow the model to converge).

For duration values, the effect of type of knowledge (*X*^2^ = 87.03, p < .0001) was significant due to longer values in the not-shared knowledge condition than in the shared knowledge condition. The effect of informational status was also significant (*X*^2^ = 20.49, p < .0001) due to longer values in the contrastive fragment condition compared to the two other conditions. The type of knowledge x informational status interaction was not significant (*X*^2^ = 2.62, p > .20). For F0 values, neither the effect of type of knowledge (*X*^2^ = 2.33, p = 0.13) nor the effect of information status (*X*^2^ = 4.68, p = .10) reached significance. The type of knowledge x informational status interaction was not significant (*X*^2^ = 0.93, p > .20). For both models there was no significant effect of the repetition when it was added to the models as a fixed factor (for duration values: *X*^2^ = 2.62, p > .20; for F0 values: *X*^2^ = 2.03).

## Discussion

The aim of the present study was to investigate whether the manipulation of shared knowledge between interlocutors impacts the prosody produced by the speaker during semi-spontaneous interactions. We were interested in two kinds of prosodic variations: global prosodic variations that affect entire utterances (i.e., pitch range and speech rate variations) and more local prosodic variations that encode informational status of words (i.e., prosodic phrasing to encode the informational status of referents). To answer this question, we used a collaborative game in which we manipulated both the presence/absence of shared knowledge between a director and an addressee and the informational status of target words.

Based on previous studies, our first hypothesis was that, in the absence of shared knowledge, participants would modify their global prosodic features to facilitate addresses’ comprehension. In line with our hypothesis, we found that directors spoke more slowly and with larger pitch excursions when they did not necessarily share knowledge on target objects with their addressee than when they did it. This result corroborates studies from clear speech literature (see [12] for a review) by showing that directors adapt their prosodic production to their addressee when they are aware of potential speech perception difficulties on their part. It is important to note that our study did not explicitly test the mechanism underlying such accommodation process. However, we can speculatively offer some possibilities. An audience design explanation of the decrease of speech rate and increase of pitch span we observed is that speakers ‘clarified’ their speech to increase addressee understanding ([12]). Within this framework, speakers in the not-shared knowledge condition recognized that their addressees needed extra help. This realization may have triggered a speaking mode similar to ‘clear speech’ that provided additional information, which would help the addressee to complete the task. According to [12] clear speech modification refers to a distinct speaking style that speakers adopt when they are aware of a speech perception difficulty on the part of the listener due to background noise, a hearing impairment, or a different native language. All clear speech research converges on the idea that clear speech typically involves a wide range of acoustic/articulatory adjustments, including a decrease in speech rate (longer segments as well as longer and more frequent pauses), wider dynamic pitch range, greater sound-pressure levels, more salient stop releases, greater rms intensity of the non-silent portions of obstruent consonants, higher-voice intensity, vowel space expansion and vowel articulation. In our study, the not-shared knowledge condition induced a decrease in speech rate and a wider dynamic pitch range which are the two main acoustic adjustments observed in case of clear speech contexts. We can thus reasonably interpret these two modifications as resulting from an adjustment the speaker made because (s)he anticipated a perception difficulty from his/her conversational partner, which is exactly the definition of “clear speech”.

However, as for the majority of clear speech studies conducted in the past, we cannot exclude that the effect of not-shared knowledge in our study is not the result of audience design per se, but rather results from production constraints on the speaker’s part (see [13–15, 17]). Specifically, not-shared knowledge between interlocutors could have slowed down the planning process of speech which results in a decrease of the speaker’s speech rate and an increase of his/her pitch excursions. Our results are thus also consistent with a mechanism in which not-shared knowledge between interlocutors complicates production processes on the speaker’s part, rather than induce adjustment of prosodic forms on the basis of a specific representation of the addressee’s needs. Thus, research must be further developed to determine whether shared knowledge manipulation affects prosody of the speaker via planning effects or not.

Regardless of the mechanism underlying the effect of shared knowledge we observed on speech rate and pitch range, our result can also be interpreted in parallel with those of Rosa, Finch, Bergeson & Arnold [38]. In their study, speakers participated in two instruction-giving experiments in which they gave instructions to listeners to move objects to locations on a board. The attention of the listener was manipulated through the number of tasks in which they were involved: in the distraction condition, addressees might experience difficulties since they were also completing a demanding secondary computer task; in the attentive condition they paid full attention to the main task. The authors found that speakers use longer words for distracted listeners compared to attentive listeners. Since in our study we found more slowly speech rate (measured as number of syllables/s) and longer durations of the last syllable of target words when knowledge was not-shared between interlocutors compared to when it was shared, our result is in line with those of Rosa, Finch, Bergeson & Arnold, by showing that speakers modify duration of speech segments when they were aware of an interlocutor’s difficulty. For Rosa, Finch, Bergeson & Arnold, this perception difficulty consisted in distraction of listeners’ attention, while in our study, this perception difficulty was due to an absence of shared knowledge between interlocutors. However, it is important to note that in our study the modification of durations of the last syllable of target words resulted from a global speech rate modification. By contrast, in Rosa, Finch, Bergeson & Arnold’s study, this duration effect seems to be localized to the target word and does not appear to affect global speech rate. However, given that the authors have not explained how speech rate was calculated, one could argue that the different results obtained could be linked to the different portion of speech segments used to define speech rate. The entire fragment was used in our study whereas smaller portions of speech segments appear to have been used in Rosa, Finch, Bergeson & Arnold’s study. Moreover, it is interesting to notice that in both studies, duration of target words did not vary depending on whether target nouns were contrastive/unpredictable vs. non-contrastive/predictable, suggesting that in these two kinds of tasks, participants did not prosodically encode informational status of words.

Indeed, in our study, the observation that directors more often produced 1 AP phrasing (between 75% and 80%) rather than 2 AP phrasing whatever the knowledge shared by participants and the informational status of words was unexpected. Specifically, when knowledge was shared, we expected participants to prosodically encode the contrastive status of target nouns by producing target nouns in a separate AP from the following adjective. We thus expected more 2 AP phrasing in the contrastive noun condition compared to the contrastive adjective condition in the shared knowledge condition. By contrast, speakers did not increase their 2 AP phrasing productions regardless of the informational status of target noun and of the knowledge shared with their addressee. This result contrasts with previous studies in the field reporting that speakers phrased the contrastive information in a separate AP when knowledge is shared between interlocutors ([7, 8, 11]).

A way to explain this result is to consider the cognitive load of the task due to visual salience effects. Interestingly, [39, 40] showed an effect of the presence of visual competitors on linguistic choices of the speakers when an addressee was present while this visual effect of the competitor disappeared in the absence of an addressee. In their experimental paradigm, they manipulated the visual saliency of the referent by presenting a picture in which a visual competitor was present or not. The authors found an effect of the visual competitor on the choice of the referring expression: speakers used fewer pronouns and more repeated noun phrases when a visual competitor was present in the scene than when there was no visual competitor. The authors concluded that visual salience effects were due to adjustments that speakers make when they speak to an addressee. [41] did not find such adjustment to the addressee. Using a referential communication task, they showed that giving an explicit focus on addressee’s perspective did not influence reference production of the speaker. While speakers were able to engage in perspective taking, they did not use this knowledge during the task. In other words, they referred to their privileged knowledge, disregarding the addressee’s perspective. In the present study, visual context always contained two target objects and a competitor regardless of the type of knowledge condition. In line with [39, 40], we hypothesized that considering the visual context of the target objects (meaning the two target objects plus the competitor) would require a lot of attention making the task cognitively demanding for the director. Interestingly, in Michelas, Faget, Portes, Lienhart, Boyer, Lançon, et al. ([11)’s study in which control speakers prosodically encoded the informational status of referents, no competitor (i.e., no object of the same type but with a different color for the given noun condition and no object of the same color but a different type for the contrastive noun condition) was present in the map for each given location. The absence of such competitors in the visual context could have made the task less cognitively demanding for speakers than in the current study. Thus, assuming a high cognitive load of our task, it could be the case that directors were not able to keep track of the salience of objects (in terms of non-contrastive/contrastive status) and thus did not prosodically encode this salience. It is interesting to note that this difficulty of speakers under cognitive load to take discourse salience into account was also found by [42]. In this study, speakers under cognitive load (they had to spread attentional resources between two tasks) had more difficulties to use pronouns to refer to less salient referents than speakers who performed only one task. Similarly, in our task, it can be the case the speakers under cognitive load had chosen the most economical linguistic forms for themselves which consisted in not using prosodic phrasing to encode informational status of target nouns. But regardless of the cognitive load of our task, it is worth noting that in 100% of cases, the participants successfully completed the interactive task. There was no case in which the task was interrupted because of great difficulties to complete it. Moreover, it is interesting to note that in only 2% of cases, the confederate put the cross in the wrong place showing that in 98% of cases, speakers succeeded in guiding the confederate to put the cross in the right place. These two points reinforce the idea that the task we have created is not a burdensome task for the participants but that it requires more attentional resources than a task such as the one used by [11] in which the participants can focus on the prosodic encoding of the informational status of referents.

Interestingly, when taking a closer look at the acoustic cues of prosodic phrasing produced by our participants, we found an effect of shared-knowledge manipulations on duration of target syllables but not on height of these syllables. This effect confirms the fact that the increase in duration we observed in the not-shared condition resulted from global speech rate manipulation and did not occur specifically on target words. Indeed, if this modification of duration has translated variations of prosodic phrasing, we would have expected that the height of the syllable increased at the same time as the duration of this one. However, this was not the case and this confirms the relevance of the criteria we used to differentiate cases in which target nouns were produced in the same AP as the following adjectives (1 AP phrasing) from cases in which they were produced in a separate AP (2 AP phrasing; see also [11] for a similar procedure). Moreover, the fact that syllable duration did not vary as a function of the informational status of target words also indicates that the increase of target duration we observed reflects global speech rate modification and not acoustic prominence to encode informational status of words.

Concerning the speaker’s global prosodic modifications we observed in the not-shared condition, one could argue that these modifications could be impacted by the fact that, in our task, the addressee was a confederate. Indeed, we know the attributions that language users have about their task partner can impact how they adapt their language ([43]) and that using a confederate in the addressee role might be risky for studies of language in dialogue contexts ([44]). For these reasons, we run an additional pilot study using exactly the same procedure as previously described except that the additional participants interacted with other naïve participants and not a confederate (see S3 Appendix). These preliminary findings are in line with the current study and suggest that participants tended to modify their global prosodic variations (i.e., pitch range and speech rate variations) when speaking to naïve addressees in the same way as they did when speaking to a confederate while they tended to not prosodically encode the informational status of referents regardless of the knowledge condition. Thus, more research is now required to test our hypothesis that if the speaker has to take into account the possible visual competitors to give relevant information to his/her addressee each time s/he prepares the prosodic encoding of referents, s/he would probably not encode the informational status of these referents. In a more general manner, a large number of previous studies considers that the difficulty of the communicative task may influence the degree to which speakers appear to be modeling their listeners (see for instance [14, 15, 38, 39, 40, 45]). Thus, the next step of this research will be to determine the constraints under which speakers could be more/less sensitive to addressee’s knowledge.

To conclude, the present study reveals that in French the speaker’s knowledge of what the addressee knows or does not know affects his/her prosodic choices. When knowledge was not shared, we found that speakers modified their global prosody in terms of speech rate and pitch span modulations. However, and unexpectedly, we also found that speaker-internal constraints seemed to affect local prosodic encoding of informational status of words production since our participants who were involved in a more cognitive demanding task than in previous studies (e.g., [11]) did not modify their AP phrasing to encode pragmatic status of referents when knowledge was shared. These results are in line with the idea that complex reasoning usually implicated in constructing a model of the addressee co-exists with speaker-internal constraints such as cognitive load of the task to affect the production of speech ([14, 15]).

## Supporting information

S1 Appendix Complete instructions given to participants. (DOCX)

S2 Appendix Extract from the dialogue between one participant (the director) and the confederate (the addressee) corresponding to the screen views shown in Fig 1. (DOCX)

S3 Appendix Pilot study including 5 additional participants that interacted with other naïve participant. (DOCX)

S1 Fig Figure shown to participants during instructions. The cross is located between the purple rake and the purple schoolbag.
(TIFF)

S2 Fig Figure shown to participants during instructions. The cross is located between the red scarf and the brown scarf.
(TIFF)
