# Supplementary material for: The impact of shared knowledge on speakers’ prosody
Source: PLoS One. 2019 Oct 14;14(10):e0223640. doi: 10.1371/journal.pone.0223640 (PMC6791546; doi:10.1371/journal.pone.0223640)
Supplement: S1 Appendix — (DOCX) [file pone.0223640.s001.docx]

**Appendix I. Instructions given to participants.**

*The confederate - who was introduced to the participants as the experimenter but was naïve about the scientific aims of the experiment - gave instructions to them.*

During this experience, we will play a game together.

We will both see different objects and different colors on our respective screens. These objects will appear on grids and we will have the same grids at the same time.

For each grid, you will see a cross on your own screen, but I won’t have this cross on my own screen. Your task is to tell me where to place the cross between different objects by telling me the type of objects and the color of objects of the two critical objects between which the cross is located.

For instance, in the following grid (see S1 Fig), the cross is located between the purple rake and the purple schoolbag.

The cross can be located vertically (such as in S1 Fig) and in this case you have to tell me the objects from left to right) or the cross can be located vertically (such as in S2 Fig) and in this case you have to tell me the objects from top to bottom).

Once I have placed the cross on my grid, we will click together on the OK button to bring up the next grid.

To make the game more difficult, in some grids there will be white boxes while in some others, there will be black boxes. In case of white boxes, you are sure that I share exactly the same objects and colors as you. But in case of black boxes, it is possible that you have either a different type of object or a different color of object as me in those boxes.

At the end of the game, we win if I have correctly placed all the crosses in the right places.

We are going to start with 6 practice grids to make sure everything is going well.

But before that, we are going to make sure that we name all the objects and the colors in the same manner. To do so, you are going to tell me what you see in the following pictures by naming the objects by their type and their color.

Do you understand? Do you have any questions?
