# Supplementary material for: The impact of shared knowledge on speakers’ prosody
Source: PLoS One. 2019 Oct 14;14(10):e0223640. doi: 10.1371/journal.pone.0223640 (PMC6791546; doi:10.1371/journal.pone.0223640)
Supplement: S2 Appendix — (DOCX) [file pone.0223640.s002.docx]

**Appendix II. Extract from the dialogue between one participant (the director) and the confederate (the addressee) corresponding to the screen views shown in Figure 1.**

Director : *ok donc là tu mets la croix entre la souris bordeau et la maison bordeau et les deux sont dans des cases noires.*

Addressee: *alors je vois bien la souris bordeau mais je n’ai pas de maison bordeau à proximité. Où est-ce que je dois mettre la croix par rapport à la souris bordeau ?*

Director : *En fait il faut que tu mettes la croix à droite de la souris bordeau ce qui fait que la croix se situe dans la deuxième colonne de la grille.*

Adresse : *ok parfait je vois. C’est bon j’ai mis la croix.*

Director: Ok then you put the cross between the red mouse and the red house, and both are in black boxes.

Addressee: Well, I can see the red mouse, but I don’t have a red house nearby. Where do I have to put the cross relative to the red mouse?

Director: You have to put the cross on the right of the red mouse so that the cross is in the second column of the grid.

Addressee: ok perfect I see. I put the cross, It's fine for me.
