# Supplementary material for: The impact of shared knowledge on speakers’ prosody
Source: PLoS One. 2019 Oct 14;14(10):e0223640. doi: 10.1371/journal.pone.0223640 (PMC6791546; doi:10.1371/journal.pone.0223640)
Supplement: S3 Appendix — (DOCX) [file pone.0223640.s003.docx]

**Appendix III. Pilot study including 5 additional participants that interacted with other naïve participants.**

**Method**

Participants: Five participants participated in the pilot experiment. None has participated in the main experiment.

Material and procedure: The material and procedure were the same as in the main experiment except that the additional participants interacted with other naïve participants and not a confederate.

**Results**

The same analyses as previously described were conducted. These analyses showed that participants increased their pitch range (154 Hz vs. 165 Hz; *X*^2^ = 5.19, p < .05) and decreased their speech rate (5.56 syl/s vs. 5.07 syl/s; *X*^2^ = 8 .00, p < .01) in the not-shared knowledge condition compared to the shared condition when speaking to naïve addressees in the same way as they did when speaking to a confederate. Moreover, in these additional analyses, the informational status of referents x type of knowledge interaction was not significant (*X*^2^ = 3.90, p = .142) showing that participants did not use more 2 APs than 1APs phrasing when the noun was contrastive (38% of items included 2 APs) compared to when the adjective was contrastive (32% of items included 2 APs) in the shared knowledge condition.
