# Supplementary figures and images for: The impact of shared knowledge on speakers’ prosody
Source: PLoS One. 2019 Oct 14;14(10):e0223640. doi: 10.1371/journal.pone.0223640 (PMC6791546; doi:10.1371/journal.pone.0223640)

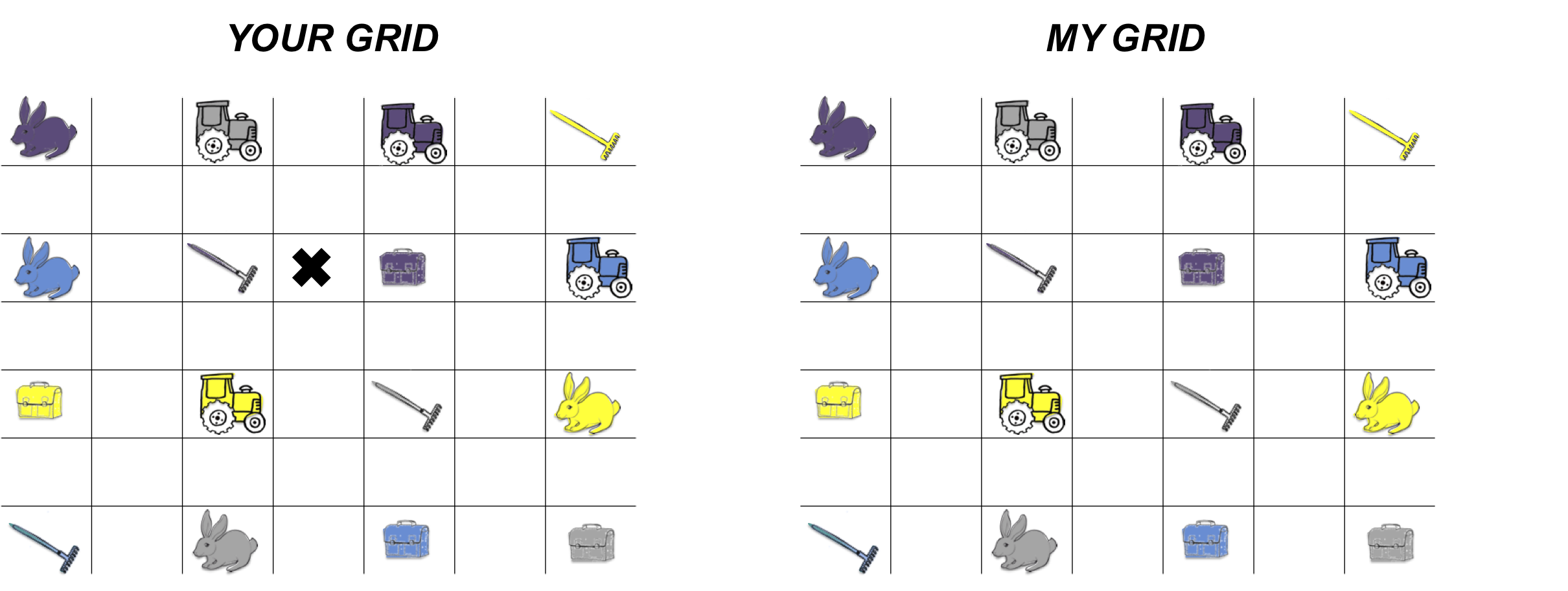

Supplement: S1 Fig — The cross is located between the purple rake and the purple schoolbag. (TIFF) [file pone.0223640.s004.tiff]

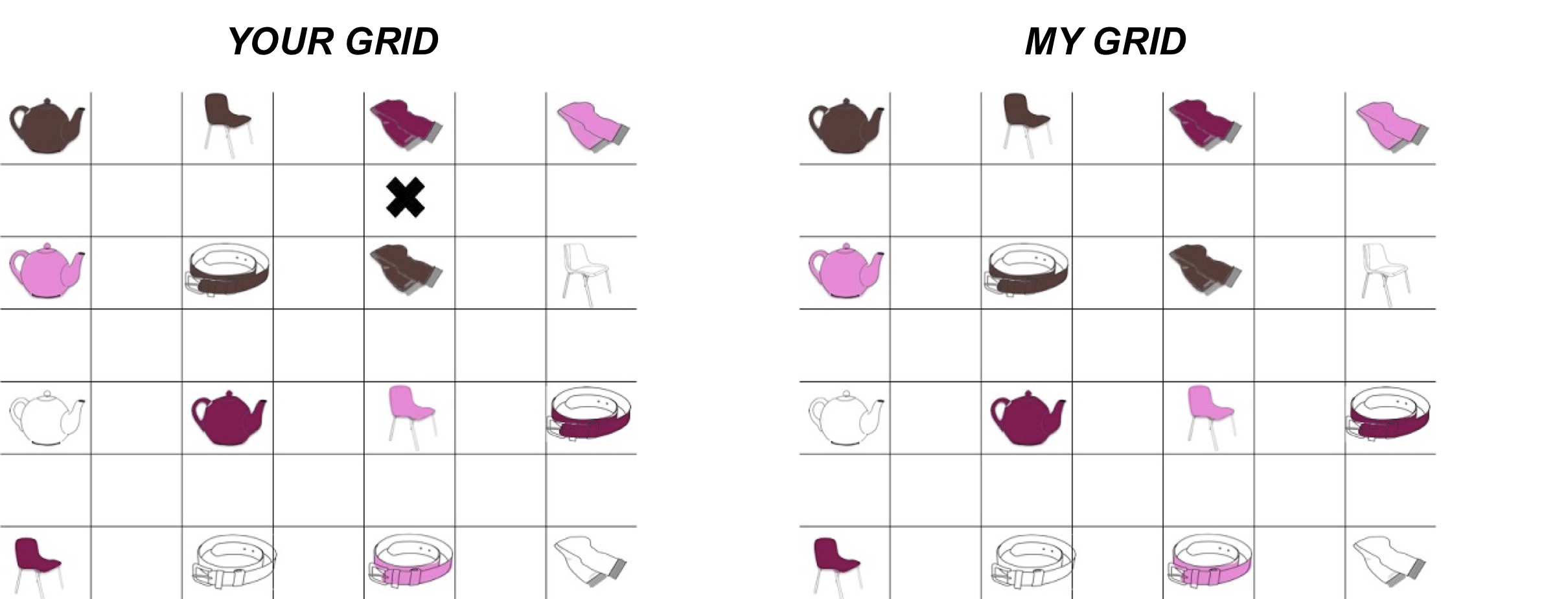

Supplement: S2 Fig — The cross is located between the red scarf and the brown scarf. (TIFF) [file pone.0223640.s005.tiff]
